# Food neophobia and its association with diet quality and weight in children aged 24 months: a cross sectional study

**DOI:** 10.1186/s12966-015-0184-6

**Published:** 2015-02-13

**Authors:** Rebecca A Perry, Kimberley M Mallan, Jasly Koo, Chelsea E Mauch, Lynne A Daniels, Anthea M Magarey

**Affiliations:** Discipline of Nutrition and Dietetics, School of Health Sciences, Flinders University, Adelaide, 5001 Australia; Institute of Health and Biomedical Innovation, School of Exercise and Nutrition Sciences, Queensland University of Technology, 60 Musk Avenue, Kelvin Grove, Brisbane, 4059 Australia

**Keywords:** Children, Neophobia, Diet, Weight, Fruit and vegetables, Discretionary foods

## Abstract

**Background:**

Food neophobia, the rejection of unknown or novel foods, may result in poor dietary patterns. This study investigates the cross-sectional relationship between neophobia in children aged 24 months and variety of fruit and vegetable consumption, intake of discretionary foods and weight.

**Methods:**

Secondary analysis of data from 330 parents of children enrolled in the NOURISH RCT (control group only) and SAIDI studies was performed using data collected at child age 24 months. Neophobia was measured at 24 months using the Child Food Neophobia Scale (CFNS). The cross-sectional associations between total CFNS score and fruit and vegetable variety, discretionary food intake and BMI (Body Mass Index) Z-score were examined via multiple regression models; adjusting for significant covariates.

**Results:**

At 24 months, more neophobic children were found to have lower variety of fruits (β = −0.16, p = 0.003) and vegetables (β = −0.29, p < 0.001) but have a greater proportion of daily energy from discretionary foods (β = 0.11, p = 0.04). There was no significant association between BMI Z-score and CFNS score.

**Conclusions:**

Neophobia is associated with poorer dietary quality. Results highlight the need for interventions to (1) begin early to expose children to a wide variety of nutritious foods before neophobia peaks and (2) enable health professionals to educate parents on strategies to overcome neophobia.

## Background

Food neophobia, the rejection of novel or unknown foods, is minimal at infancy and peaks sometime between two and six years [1]. A lower level at infancy is adaptive to facilitate the introduction of new foods [2]. The peak in food neophobia occurs when children start to explore their surroundings outside parental guidance, thereby functioning as a protective mechanism when risk of consumption of toxic or poisonous food is high [1,2]. Neophobia is not a permanent dislike for new foods; acceptance can be promoted through repeated exposure to or modelling the consumption of the rejected food(s) [2-4]. Neophobia overlaps to some extent with the broader concept of ‘picky/fussy’ eating which refers to the rejection of both familiar and unfamiliar foods and may not be as easy to overcome through repeated exposure [1,5]. It is worth noting that measurement of both neophobia and picky/fussy eating by parents reflects parental perceptions of the child’s behaviour. In particular, the rejection of foods a child normally eats (‘fussy eating’) may in fact reflect lack of appetite (i.e., fullness) rather than fussy eating behaviour. The present study focuses specifically on the rejection of novel foods in order to assess correlates of neophobia as it begins to emerge in toddlerhood. In the current environment of a plentiful, safe but obesogenic food supply, food neophobia is maladaptive and, along with our known predisposition to prefer sweet and salty flavours, merely serves to reduce overall diet quality and variety [2].

In Australia, many children do not meet the recommendations for fruit and vegetable intakes; 61% and 22% of four to eight year olds meet fruit and vegetable guidelines respectively [6]. This warrants concern as children may forgo the potential health benefits of fruits and vegetables, such as protection against chronic diseases [7,8] and adiposity [9,10]. The development of these eating habits involves a complex interplay between genetic and environmental factors [11]. Current literature suggests that neophobia may be a contributing factor, through hindering the acceptance of new foods differentially. In particular, more neophobic children are found to have lower preferences for and intake of both fruits and vegetables [12-15].

In addition to making recommendations regarding the quantity of fruits and vegetables to consume, dietary guidelines encourage children to consume a variety of ‘colours’ from these groups due to the variation in nutrients provided by the different fruits and vegetables [16]. Despite the aforementioned evidence, there has been little research regarding the impact of neophobia on the variety of fruit and vegetables consumed. In a brief review [17] it was suggested that the decline in fruit and vegetable variety observed during the third year of life [18] coincides with the emergence of the developmental phase of neophobia. In a study that measured self-reported food variety via a food choice questionnaire vegetable variety was negatively related to food neophobia in a sample of (n = 339) 4–22 year olds [19].

Discretionary food, defined as energy-dense and nutrient-poor foods by the Australia Guide to Healthy Eating, is another food group of concern in children’s diets [16]. In Australia, the proportion of total energy provided by discretionary foods increases with age, ranging from 24% for two to three year olds to 38% for 14 to 16 year olds, exceeding the recommended limit of 5 to 20% in all age groups [20]. Few studies have explored the relationship between neophobia and discretionary food intake. A study of four to five year olds in the UK found no association between neophobia and intake of snack foods at a test lunch at school [14]. Similarly, a study of two to six year olds in the UK found no association between neophobia and intake of sweet/fatty snack foods [21]. Given that discretionary foods have been associated with excess energy intake, and consequently weight gain and obesity [22] there is a need to explore the relationship between neophobia and discretionary foods further.

The potential influence of neophobia on dietary intake may translate into unhealthy weight outcomes in children at both ends of the spectrum, i.e. underweight and obesity. Current literature exploring this relationship presents contradictory results. Of four studies, two found no association [23,24], one observed overweight/obese children to be more neophobic [25], and another found that significantly more children classified as ‘picky’ were underweight compared with the control group [26]. These contradictory results may be attributed to differences in demographic characteristics of the sample (including child age) and recruitment techniques. With inconclusive evidence, more research is required to ascertain if neophobia is a significant predictor of weight. The aim of this study was to investigate the associations between neophobia and variety of fruits and vegetables consumed, the intake of discretionary foods and weight outcomes in a sample of Australian children aged 24 months.

## Methods

### Study design

This is a secondary analysis of data collected from the control group of the NOURISH randomized controlled trial (RCT) and the South Australian Infant Dietary Intake (SAIDI) study. The NOURISH RCT tested the effectiveness of an early feeding intervention promoting positive feeding practices (Australasian Clinical Trials Registration ACTRN 1260800056392) [27]. SAIDI was a concurrent longitudinal study tracking the dietary intake of South Australian children [28].

### Recruitment and data collection

Both studies followed the same recruitment, assessment and dietary intake protocols, which have been described [27,28]. In brief, recruitment was carried out between March 2008 and April 2009 in two stages. In Stage 1, a consecutive sample of mothers delivering healthy infants (≥37 weeks gestation, ≥2500 g) was approached for permission to be re-contacted. Three to six months later, at Stage 2, written informed consent was obtained for full enrolment in the study. Mothers in the NOURISH study were first-time mothers recruited from both Adelaide and Brisbane maternity hospitals. Mothers, regardless of parity, were also recruited for SAIDI from hospitals in metropolitan and regional South Australia. Collection of data occurred at birth and mean child ages5.2 (± SD = 1.3) months, 14.1 (± SD = 1.1) months and 24.0 (± SD = 0.7) months.

Ethics approval was obtained from 11 Human Research Ethics Committees covering Queensland University of Technology, Flinders University and all the recruitment hospitals (Queensland University of Technology HREC 00171 Protocol 0700000752). Written informed consent was obtained from all families.

### Child and maternal socio-demographic covariates

Within 72 hours of the birth of their infant, consenting mothers completed a questionnaire providing socio-demographics, including age, education level and marital status. Child gender and maternal parity were collected from medical records. Data collected at child ages 5, 14 and 24 months included age solids first introduced and breastfeeding duration. Breastfeeding duration was categorised as: never; less than 6 months; 6–12 months, and more than 12 months.

### Child food neophobia

Neophobia was measured at child age 24 months using the Child Food Neophobia Scale (CFNS); a validated tool that has shown good internal consistency (Cronbach’s alpha of 0.91) [29]. Of the original 10 items, four items were excluded for not being age-appropriate (e.g. my child likes to eat in ethnic restaurants). The six remaining items were: *My child does not trust new foods; If my child doesn’t know what’s in a food, s/he won’t try it; My child is afraid to eat things s/he has never tried before; My child will eat almost anything (reversed score); My child is very particular about the food s/he will eat, and My child is constantly sampling new and different foods (reversed score)*. These six items showed good internal consistency for the present sample with a Cronbach’s alpha of 0.91. Responses were measured using a four-point scale, ranging from ‘strongly disagree’ to ‘strongly agree’. Total CFNS score was computed, with a range of six to 24. Higher scores indicated a greater parental perception of food neophobia.

### Dietary intake

Dietary intake data collected at child age 24 months was included in the present paper, and comprised a 24-hour recall and a two-day diet record. All mothers were provided with a food record booklet, a second booklet to provide to other carers of the child, measuring sheet with life-size images of spoon, cup and bottle sizes, and a set of measuring spoons to assist with keeping accurate food records. The 24-hour recall was collected from mothers via an unannounced phone interview by a dietitian, using the multiple pass methodology [30]. Mothers were unaware that a dietitian was conducting the recall. To maximise successful contact, mothers were asked to identify unsuitable times to be called. During the recall, recipes with ingredient quantities and amount eaten were obtained for home prepared dishes. Breastfeeds were recorded as time the child spent suckling and quantified as 10 g/min to a maximum of 10 min per feed [31]. At conclusion of the 24-hour recall, mothers were allocated two specific days to record their child’s food and beverage intake. Days were chosen to ensure two weekdays and one weekend day were included in the three-day intake data for each child. Completed records were returned via reply paid envelope.

Data were entered by dietitians into FoodWorks Professional version 9, using the AUSNUT 2007 database from the National Children’s Nutrition and Physical Activity Survey [32]. Nutrient information of infant food and formula products not available in the database were sourced from websites, food companies or the nutrient information panel on the product packaging. Home prepared dishes with more than five ingredients were entered as recipes. A comprehensive protocol for checking FoodWorks data entry was developed and implemented. All foods in FoodWorks are assigned a unique 8-digit code which allows categorization of foods into food groups and sub-groups [32]. Additional items were assigned an appropriate code by study investigators and managed by a single dietitian. The resulting database was exported into SPSS for analysis.

Variety was defined as the number of different sub-groups (within a major food group) a child ate over three days. Fruit and vegetable total variety scores were defined separately, with a total of 30 and 36 sub-groups identified respectively. A child was given a point for each fruit/vegetable sub-group from which they ate, regardless of the frequency or quantity eaten and then the points summed to give a total variety score. A minimum quantity was not imposed as measuring exposure to a variety of fruits and vegetables was more important in this age group. Different types of the same fruit/vegetable did not constitute different varieties. For example, red and green apples were considered a single sub-group of ‘apples’.

Discretionary foods were defined by the Australian Guide to Healthy Eating 2013 [16] and generally are foods with high energy and low nutrient density (excluding fats and oils). Contribution of discretionary foods, as percentage of average daily energy intake over three days, was used as an outcome variable.

### Anthropometric data

Children were weighed with outer clothing removed (clean nappies, underwear and singlets were permitted) by trained study staff at assessment clinics or home visits. In the case of families living in regional South Australia, the primary carer was instructed to have the child weighed and measured by a doctor or child health nurse at a local clinic (it was unfeasible to have these families attend assessment clinics in Adelaide due to long travel times). Weight was taken to nearest 10 g using baby scales at child age 5 months and using standing scales at 24 months. Length was measured to nearest 0.5 cm using a measuring mat at child age 5 months and height was measured to the nearest 0.5 cm using a stadiometer at 24 months. Weight-for-age and Body Mass Index (BMI) Z-scores were calculated at child age 5 and 24 months, respectively, using World Health Organisation (WHO) 2006 Growth Standards using the WHO Anthro software program version 3.0.1 and macros [33]. At child age 5 months mothers were weighed and measured (without shoes) and maternal BMI calculated. Around 25% of SAIDI mothers living in regional areas of South Australia self-reported their current weight and height at child age 5 months. Travel to regional areas was not feasible within the budget constraints of the study.

### Data analysis

Only NOURISH control participants (n = 186) and SAIDI participants (n = 172) with all three days of food intake data at child age 24 months were considered for the present study (total n = 358). Due to missing data on some covariates the final sample size included in the multiple linear regression models was reduced to n = 330.

Data analysis was performed using IBM SPSS Version 22. The four ‘outcome’ variables of interest (fruit variety, vegetable variety, percentage of energy from discretionary foods and child BMI Z-score) were normally distributed. From the maternal and child characteristics presented in Table 1, covariates were selected for inclusion in the regression models based on a significant association (p ≤ 0.05) with at least one of the outcome variables. Marital status was not considered given that most of the mothers in the sample were partnered. Four hierarchical multiple linear regression models were used to investigate the relationship between child food neophobia (total CFNS score) and each of the ‘outcome’ variables. The significant covariates (maternal age, other children, education, [maternal] BMI and child weight-for-age Z score at age 5 months) were entered at Step 1 and CFNS score was added at Step 2. Multi-collinearity was checked using collinearity diagnostics and Cook’s distance was used to screen for influential data points (of which there were none). A significance level of p ≤ 0.05 was applied for the final multiple regression models.

**Table 1:** **Characteristics of mother-child dyads (n = 358** ^**a**^ **)**

**Demographic variable**	**Mean ± SD or%(n)**
**Maternal characteristics**	
Age at delivery (years)	31.6 ± 4.8
BMI at T1 (kg/m^2^)	26.1 ± 5.8
Marital status (partnered)	96.9 (346)
Mother’s education level (university degree)	57.0 (204)
Other children (none)	66.9 (237)
**Child characteristics**	
Gender (boy)	45.8 (164)
Breastfeeding duration	
Never breastfed	1.7 (6)
Less than 6 months	28.3 (101)
6 to 12 months	29.4 (105)
More than 12 months	40.6 (145)
Age of introduction of solids (weeks)	21.9 ± 5.2
Weight for age Z-score at T1^b^	−0.3 ± 1.0
Total Child Food Neophobia Score^c^	12.8 ± 3.6

^a^N values range from 344 to 358 due to missing data.

^b^Calculated at infant age M = 5.2, SD = 1.3 months using WHO Anthro software program version 3.0.1 and macros [33].

^c^Total score on Child Food Neophobia Scale [29], 6 items, 4 point scale, higher scores indicative of stronger behavioural display of neophobia; reported by mother at child age M = 24.0, SD = 0.7 months.

## Results

Characteristics of mother-child dyads are presented in Table 1. The majority of mothers were partnered and more than half had completed a university degree. Over two-thirds (70%) of mothers in the sample breastfed their child for longer than 6 months.

Unstandardized regression coefficients (B) with standard errors (SE) and standardised regression coefficients (β) for the final hierarchical regression models are shown in Table 2. The regression model for fruit variety over three days was significant (R^2^ = 0.12; R^2^_adj_ = 0.10; F(6, 324) = 7.21, p = 0.003). Children with a higher total CFNS score (β = −.16, p = .003) and those with siblings (β = −0.12, p = 0.04) were likely to have a lower variety of fruits in their diet. Children whose mother was older at birth (β = 0.17, p = 0.003) and university educated (β = 0.21, p < 0.001) were likely to have a wider variety of fruits in their diet.

**Table 2:** **Hierarchical multiple regression analyses of relationship between child food neophobia and fruit variety, vegetable variety, discretionary food intake and weight (child age M = 24.0 ± SD = 0.7 months; n = 330), controlling for significant covariates**

	**Fruit variety score** ^**a**^		**Vegetable variety score** ^**b**^		**Percent energy from discretionary foods** ^**c**^		**BMI Z-score** ^**d**^	
Mean ± SD	4.2 ± 2.1		4.9 ± 2.9		18.4 ± 10.2		0.8 ± 1.0	
	B (SE)	β	B (SE)	β	B (SE)	β	B (SE)	β
*Step 1*	ΔR^2^ = 0.093**		ΔR^2^ = 0.046*		ΔR^2^ = 0.041*		ΔR^2^ = 0.16**	
Maternal age at delivery	0.073 (0.024)	0.17*	0.12 (0.033)	0.21**	−0.23 (0.12)	−0.107	−0.021 (0.012)	−0.099
Maternal BMI at T1	−0.026 (0.019)	−0.071	−0.017 (0.026)	−0.034	0.079 (0.10)	0.045	0.024 (0.0090)	0.14*
Maternal education (not university vs university)	0.88 (0.23)	0.21**	0.59 (0.31)	0.10	−0.89 (1.16)	−0.043	0.027 (0.11)	0.013
Other children (no vs yes)	−0.52 (0.25)	−0.12*	−0.59 (0.34)	−0.097	3.60 (1.25)	0.17*	−0.077 (0.12)	−0.035
Infant weight-for-age Z-score^e^	−0.071 (0.11)	−0.034	−0.047 (0.15)	−0.016	0.72 (0.56)	0.070	0.35 (0.054)	0.34**
*Step 2*	ΔR^2^ = 0.025*		ΔR^2^ = 0.082**		ΔR^2^ = 0.013*		ΔR^2^ = 0.004	
Total CFNS score^f^	−0.092 (0.030)	−0.16*	−0.23 (0.041)	−0.29**	0.32 (0.15)	0.11*	−0.017 (.015)	−0.061
*Final model*	R^2^ = 0.12**		R^2^ = 0.13**		R^2^ = 0.054*		R^2^ = 0.17**	

*p ≤ 0.05; **p < 0.001; B = unstandardized regression coefficient, β = standardized regression coefficient (values are from the final model).

^a^Number of different fruit subgroups eaten over three days (30 fruit sub-groups in total).

^b^Number of different vegetable subgroups eaten over three days (35 vegetable sub-groups in total).

^c^Average energy contributed by discretionary foods as a percentage of average daily energy intake over three days

^d^Calculated at child age M = 24.0, SD = 0.7 months using WHO Anthro software program version 3.0.1 and macros [33].

^e^Calculated at child ageM = 5.2, SD = 1.3 months using WHO Anthro software program version 3.0.1 and macros [33].

^f^Total score on Child Food Neophobia Scale [29], 6 items, 4 point scale, higher scores indicative of stronger behavioural display of neophobia; reported by mother at child age M = 24.0, SD = 0.7 months.

The regression model for vegetable variety over three days was significant (R^2^ = 0.13; R^2^_adj_ = 0.11; F(6, 324) = 7.92, p < 0.001). Children with a higher total CFNS score (β = −0.29, p < 0.001) were more likely to have a lower variety of vegetables in their diet. Also, those whose mothers were older at birth (β = 0.21, p < 0.001) were more likely to have a wider variety of vegetables in their diet. There were non-significant trends toward those with siblings being more likely to have a lower vegetable variety score (β = −0.10, p = 0.08), and those whose mothers were university educated being more likely to have a higher vegetable variety score (β = 0.10, p = 0.06).

The proportion of daily energy intake provided by discretionary foods over three days was significantly accounted for by the final model (R^2^ = 0.054; R^2^_adj_ = 0.037; F(6, 324) = 3.09, p = 0.006). Children with a higher total CFNS score were more likely to have a higher proportion of daily energy intake provided by discretionary foods (β = 0.11, p = 0.04). Children with siblings had a higher proportion of average daily energy intake accounted for by discretionary foods (β = 0.17, p = 0.004). There was a non-significant trend toward a higher proportion of average daily energy intake accounted for by discretionary foods being consumed by children of younger mothers (β = −0.11, p = 0.07).

The regression model for BMI Z-score was significant (R^2^ = 0.17; R^2^_ad_ = 0.15; F(6, 324) = 10.70, p < .001). However CFNS score did not significantly predict BMI Z-score (β = −0.061, p = 0.24). Children who had higher weight-for-age Z-score at 5 months (β = 0.34, p < 0.001) and whose mothers had a higher BMI (β = 0.14, p = 0.008) were more likely to have a higher BMI Z-score at 24 months. There was a non-significant trend toward lower BMI Z-scores in those with older mothers (β = −0.10, p = 0.07).

## Discussion

This study aimed to examine the cross-sectional relationship between neophobia at 24 months and the variety of fruits and vegetables consumed, discretionary food intake and child weight. Results indicated that neophobia was related negatively to fruit and vegetable variety but positively related to discretionary foods intake at 24 months. Neophobia was not related to child weight at 24 months.

The key finding is that children with higher levels of neophobia are likely to consume a lower variety of fruit and vegetables. Literature examining the influence of neophobia on fruit and vegetables has mostly concentrated on preferences for these foods and the quantity eaten. Three of seven [14,15,34] and four of seven studies [14,15,34,35] on intake found negative relationships with fruit and vegetables respectively. Studies examining preferences also reported a negative relationship with neophobia [12,13,36]. Therefore, it appears that neophobia is related to not only lower preferences for and lower quantity eaten but also lower variety of fruit and vegetables. Dietary variety is a key characteristic of dietary quality and recent dietary guidelines refer to a choosing a wide variety of foods from each food group [16].

In the present study we showed that discretionary foods contributed a larger proportion of daily energy in more neophobic children. This contradicts findings from other studies, which have found no significant relationship. This may be explained by discretionary foods being defined differently. Cooke et al. [21] measured frequency of consumption of discretionary foods (‘sweet/fatty snacks’) in 2–6 year olds using a Food Frequency Questionnaire. Discretionary food intake was represented by energy intake from chocolate biscuits and cheese crackers offered at three test meals in another study with 4–5 year olds [14]. Neither of these measures may fully capture intake over the full range of discretionary foods. Findings from our study add to the literature by including energy intake from a potentially more comprehensive range of discretionary foods based on actual intake records rather than a predefined ‘list’ of discretionary foods. Hence, this may account for the observed positive relationship found in this, but not in previous studies.

A possible explanation for the relationship between neophobia and variety of fruits and vegetables and discretionary foods is that novel foods are rejected based on the visual domain [1]. In the hunter-gatherer days, plant foods posed a significant risk of poisoning [37]. Hence, novel plant foods may incite a larger fear reaction as they do not ‘look right’ [1]. An alternative explanation is that choices are made based on food value [37]. Children are in a stage of rapid growth hence, acceptance of foods of higher energy or protein values may be more adaptive [37]. Coupled with an innate predisposition towards sweet and salty tastes [2], and the increasing exposure to discretionary foods at a young age, children may more readily accept discretionary foods because of their higher energy content and sweet or salty flavour.

Taken together, more neophobic children appear to have a poorer eating pattern, with a lower variety of fruits and vegetables and higher energy intake from discretionary foods. It is well-established that high intake of fruits and vegetables can protect against chronic disease and adiposity [7-10]. These health benefits can be maximised through consuming a variety of fruits and vegetables [16]. On the other hand, discretionary foods have been proposed to displace core foods (which provide essential nutrients for optimal health) and contribute to excess energy intake, weight gain and obesity [38]. Therefore, the present findings warrant concern about the potential relationship between food neophobia and children’s dietary quality.

The failure to find a relationship between neophobia and weight outcomes may be due to the fact that two years of age is too early for the potential impact of poorer dietary on weight to manifest in neophobic children. Additionally, neophobia is assumed to share a linear relationship with BMI Z-score in our regression analysis. However, neophobia may share a curvilinear relationship i.e. neophobia may be related to both overweight and underweight. In this case, a logistic regression would be more appropriate to identify this relationship. This could not be conducted with the present sample as the analysis would have been underpowered to detect any difference, with only one child in the underweight category.

To our knowledge, this study is the first to investigate the relationship between neophobia and fruit and vegetable variety. Also, it is the first Australian study investigating neophobia in relation to diet quality and weight outcomes in children aged two years. Another strength of the study is the relatively large sample size. In addition the use of three days of intake data (including both two weekdays and a weekend day) gives a more accurate representation of fruit and vegetable variety and energy intake from discretionary foods by taking into account day-to-day variability.

Our findings should be interpreted in light of study limitations. First, neophobia and its relationship with fruit and vegetable variety, discretionary foods and weight outcomes were investigated cross-sectionally. Hence, a causal relationship cannot be determined. Nevertheless the present results support future work in this area that specifically considers a causal pathway between neophobia and diet quality later in childhood. Second, mothers’ reports of neophobia and dietary intake were used. Interpretations of neophobic behaviours may vary among mothers. Reports of dietary intake are susceptible to social desirability bias and a drive for consistency in answers, hence reducing the accuracy of reported food intake. Third, the sample comprised predominantly well-educated mothers, who may have better knowledge of nutrition. Data from the Australian Bureau of Statistics shows that around 40% of Australian women aged between 30 and 34 years hold a bachelor degree, compared to almost 60% in the present study [39]. Mothers involved in the present study were also more likely to be in a relationship (married or de facto) than the broader Australian public (97% compared with just under 60% of Australian adults aged 25 to 34 years) [40]. This limits generalizability of results to the broader population. This may also explain why the average proportion of daily energy contributed by discretionary foods (median 19.4, IQR 12.8:27.5, n = 358) was slightly lower than the 2007 Australia National Children’s Nutrition and Physical Activity’s levels of 24% in two to three year olds [20].

Research has shown that an increased number of exposures, up to 10 to 15 [4,41,42] can transform initial rejection of new foods into acceptance in children of this age group. Studies have found both teacher [43] and parental modelling [44] to promote acceptance of novel food, especially if models are consuming foods of a similar colour [3] and it is done enthusiastically [43]. Parents have a high degree of control over exposure to food types and food-related experiences of children aged two years [11]. Hence, given the potential association between neophobia and diet quality suggested by the findings of our study, there is a need for interventions to target parents to encourage acceptance of novel nutritious foods into their regular diet. Health professionals can play an important role in educating parents on understanding neophobia as a developmentally normal stage but also equipping them with strategies such as repeated neutral exposure and modelling through which they can encourage their child to try (and eventually accept) novel foods. Early intervention would be ideal as eating habits established by five years can persist through adolescence into adulthood [45-48].

## Conclusion

In summary, this study provides evidence that more neophobic children were found to have lower dietary quality in terms of lower variety of fruits and vegetables but higher energy intake from discretionary foods. The absence of a relationship between neophobia and weight may be attributed to the age of the children in this study, and therefore the association between neophobia and weight warrants further investigation as additional follow-up anthropometric data becomes available. Results suggest the need for early interventions to encourage a wide variety of nutritious foods before neophobia peaks.

## Abbreviations

CFNS: Child food neophobia scale
SAIDI: South Australian infant dietary intake study
BMI: Body mass index
